# Cytotoxicity, oxidative stress, and genotoxicity in human hepatocyte and embryonic kidney cells exposed to ZnO nanoparticles

**DOI:** 10.1186/1556-276X-7-602

**Published:** 2012-10-30

**Authors:** Rongfa Guan, Tianshu Kang, Fei Lu, Zhiguo Zhang, Haitao Shen, Mingqi Liu

**Affiliations:** 1 Zhejiang Provincial Key Laboratory of Biometrology and Inspection and Quarantine, China Jiliang University, Hangzhou 310018, People's Republic of China; 2 College of Biological and Environmental Engineering, Zhejiang University of Technology, Hangzhou, 310014, People's Republic of China; 3 Food Science Institute, Zhejiang Academy of Agricultural Sciences, Hangzhou, 310021, People's Republic of China; 4 Zhejiang Province Center for Disease Prevention and Control (ZJCDC), HangZhou, 310051, People's Republic of China

**Keywords:** ZnO nanoparticles, Cytotoxicity, Oxidative stress, Human hepatocyte, Human embryonic kidney cells

## Abstract

Traces of zinc oxide nanoparticles (ZnO NPs) used may be found in the liver and kidney. The aim of this study is to determine the optimal viability assay for using with ZnO NPs and to assess their toxicity to human hepatocyte (L02) and human embryonic kidney (HEK293) cells. Cellular morphology, mitochondrial function (MTT assay), and oxidative stress markers (malondialdehyde, glutathione (GSH) and superoxide dismutase (SOD)) were assessed under control and exposed to ZnO NPs conditions for 24 h. The results demonstrated that ZnO NPs lead to cellular morphological modifications, mitochondrial dysfunction, and cause reduction of SOD, depletion of GSH, and oxidative DNA damage. The exact mechanism behind ZnO NPs toxicity suggested that oxidative stress and lipid peroxidation played an important role in ZnO NPs-elicited cell membrane disruption, DNA damage, and subsequent cell death. Our preliminary data suggested that oxidative stress might contribute to ZnO NPs cytotoxicity.

## Background

ZnO NPs have at least one dimension in the range of 1 to 100 nm. As compared to the ordinary ZnO powder, ZnO nanoparticle is a new type of high-functional fine inorganic material with higher chemical activity, extremely strong oxidation resistance, corrosion resistance, photocatalysis, unique stronger absorption, and shielding ability to the ultraviolet rays [1,2]. It has been widely used in consumer and industrial products, especially in cosmetics, food additives, photoelectricity, and rubber industry [3-5]. It is clear that with decreasing particle size, small particles can easily accumulate and migrate deeply in body. For these reasons information about the safety and potential hazards of ZnO NPs is required.

ZnO NPs is considered as one of the most toxic NPs with the lowest LD50 value among the engineered metal oxide nanoparticles [6]. To date several studies provided ample evidence that ZnO NPs distributed mainly in the blood, lungs, kidneys, spleen, pancreas or other organs, and bone. *In vitro* cell line studies have shown decreased mitochondrial function and oxidative stress after exposure to ZnO NPs in human embryonic lung fibroblasts (HELF) cells [7], human epidermal (A431) cells [8], human colon carcinoma (LoVo) cells [9], human lung bronchial epithelial (BEAS-2B) cells [10], hepatocellular carcinoma (SMMC-7721) cells [11], and human osteoblast cancer cell line [12]. Thus, examination of the ability of ZnO NPs to penetrate the liver and kidney is warranted.

Our objectives in this study were to determine the optimal viability assay for using with ZnO NPs in order to assess their toxicity to the liver and kidney cells. In this paper, we have evaluated the toxicity of ZnO NPs and analyzed cellular morphology, cellular viability, oxidative stress, and DNA damage in ZnO NPs-treated cells.

## Methods

### Cell culture and treatment

L02 cells (CBCAS, Shanghai, China) were cultured in RPMI 1640 medium (Gibco BRL, MD, USA); while HEK293 cells (CBCAS, Shanghai, China) in DMEM medium (Gibco BRL, MD, USA), with fetal calf serum (10%), l-glutamine (2.9 mg·mL^−1^), streptomycin (1 mg·mL^−1^), and penicillin (100 units·mL^−1^). The cells were cultured at 37°C in water-saturated air supplemented with 5% CO_2_. Culture media were changed every 2 days. Cells were passaged thrice a week. At 85% confluence, the cells were harvested using 0.25% trypsin and were subcultured into 75 cm^2^ flasks, 6-well plates, 24-well plates, or 96-well plates according to the selection of experiments.

After the monolayer of cells was placed in 6, 24, or 96-well plates, the cells were treated with a range of concentrations of nano-sized ZnO particles suspended in medium without serum for 24 h. After the 24 h treatment, the various toxicity end points were evaluated in control and ZnO particles-exposed cells.

### Cell morphology

L02 cells and HEK293 cells were exposed as mentioned above at various concentrations of ZnO NPs for 24 h. After completion of the exposure period, the cells (control and nano-ZnO exposed) were washed with phosphate buffered solution (PBS) and observed by phase contrast inverted microscopy at ×200 magnification.

### Cell activity

Mitochondrial function was evaluated by 3-(4,5-dimethylazol-2-yl)-2,5-diphenyltetrazolium bromide (MTT) assay. The MTT assay helps in cell viability assessment by measuring the enzymatic reduction of yellow tetrazolium MTT to a purple formazan, as measured at 570 nm using enzyme-labeled instrument (Tecan Co., Weymouth, UK).

### Oxidative stress

Cells were cultured in 75 cm^2^ culture flask and exposed to ZnO NPs (5 to 100 μg·mL^−1^) for 24 h. After exposure, the cells were harvested in chilled PBS by scraping and washed twice with 1 × PBS at 4°C for 6 min at 1,500 rpm. The cell pellet was then sonicated at 15 W for 10 s (3 cycles) to obtain the cell lysate.

Oxidative stress markers (malondialdehyde, MDA; glutathione, GSH; superoxide dismutase, SOD) were estimated by Nanjing Jiancheng Bioengineering Institute (Nanjing, China) according to manufacturer's protocol. Protein content was measured by the method of Lowry [13] using BSA as the standard.

### Comet (single cell gel electrophoresis) assay

DNA damage by ZnO NPs was further studied using comet assay. After treatment with nano-sized ZnO particles for 4, 12, and 24 h, the cells were rinsed with ice-cold 1 × PBS and trypsinized. Then the cells were washed once in ice-cold 1 × PBS and resuspended at 1 × 10^5^ cells mL^−1^ in ice-cold 1 × PBS. An aliquot of 10-μL cell suspension was mixed with 100 μL molten agarose (at 37°C), and 75 μL of this mixture was immediately applied to a glass slide. The slide was held horizontal at 4°C for 30 min to improve adherence. Then the slide was immersed in cold lysis solution to lyse the cells. After 50 min at 4°C in the dark, the slide was immersed in an alkaline solution (300 mM NaOH, 1 mM EDTA, pH > 13) at room temperature in the dark to denature the DNA. After 30 min the slide was placed on a horizontal electrophoresis unit, and the unit was filled with fresh buffer (300 mM NaOH, 1 mM EDTA, pH > 13) to cover the slide. Electrophoresis was conducted at 27 V (300 mA) for 40 min at 4°C in the dark. The slide was then washed gently with distilled water and immersed in 70% ethanol for 5 min. After the slide was air dried, 50 μL of ethidium bromide working solution was applied to each circle of dried agarose. All steps described above were conducted under yellow light to prevent additional DNA damage.

The slides were viewed using an epifluorescence Leica DMI 4000B microscope (Leica Microsystems Ltd., Hong Kong, China) equipped with a fluorescein filter. Observations were made at a final magnification ×400. Thirty randomly selected cells per experimental point were imaged and analyzed using CASP software (download from http://www.casp.of.pl/). Results were reported as tail moment, a parameter describing the number of migrated fragments, and represented by the fluorescence intensity in the tail, expressed as the mean of the 50 cells.

### Statistical analysis

The data were expressed as mean ± standard deviation of three independent experiments. The data was subjected to statistical analysis by one-way analysis of variance followed by Dunnett's method for multiple comparisons. A value of *p* < 0.05 was considered significant. SPSS 16.0 software was used for the statistical analysis.

## Results

### Characterization studies

**Figure 1:**
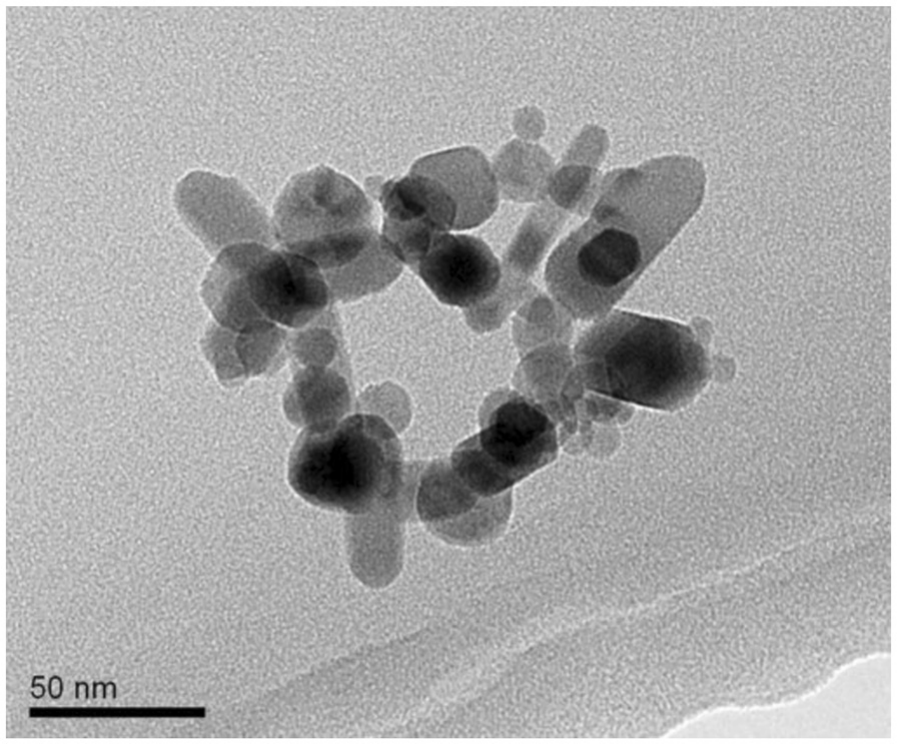
Transmission electron micrograph image of ZnO nanoparticles.

The average size of ZnO NPs (Sigma-Aldrich, St. Louis, MO, USA) as measured by transmission electron microscope (TEM) (JEOL JEM-2100, JEOL Ltd., Tokyo, Japan) was approximately 50 nm. Figure 1 shows a representative TEM image recorded ZnO nanoparticles.

### Cell morphology

**Figure 2:**
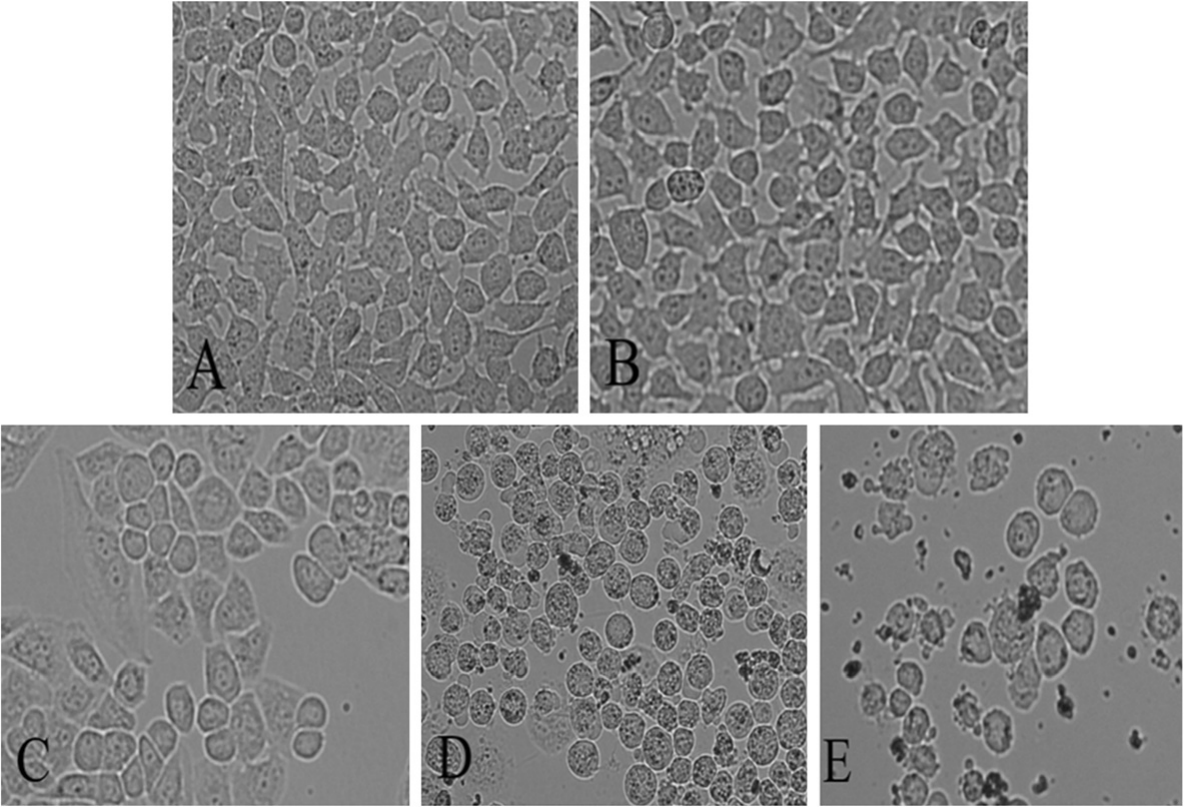
**Morphological changes of L02 cells exposed at various concentrations of ZnO NPs for 24 h.** (**A**) normal and non-ZnO NPs-treated, (**B**) 10 μg·mL^−1^, (**C**) 25 μg·mL^−1^, (**D**) 50 μg·mL^−1^, and (**E**) 100 μg·mL^−1^. Magnification ×200.

**Figure 3:**
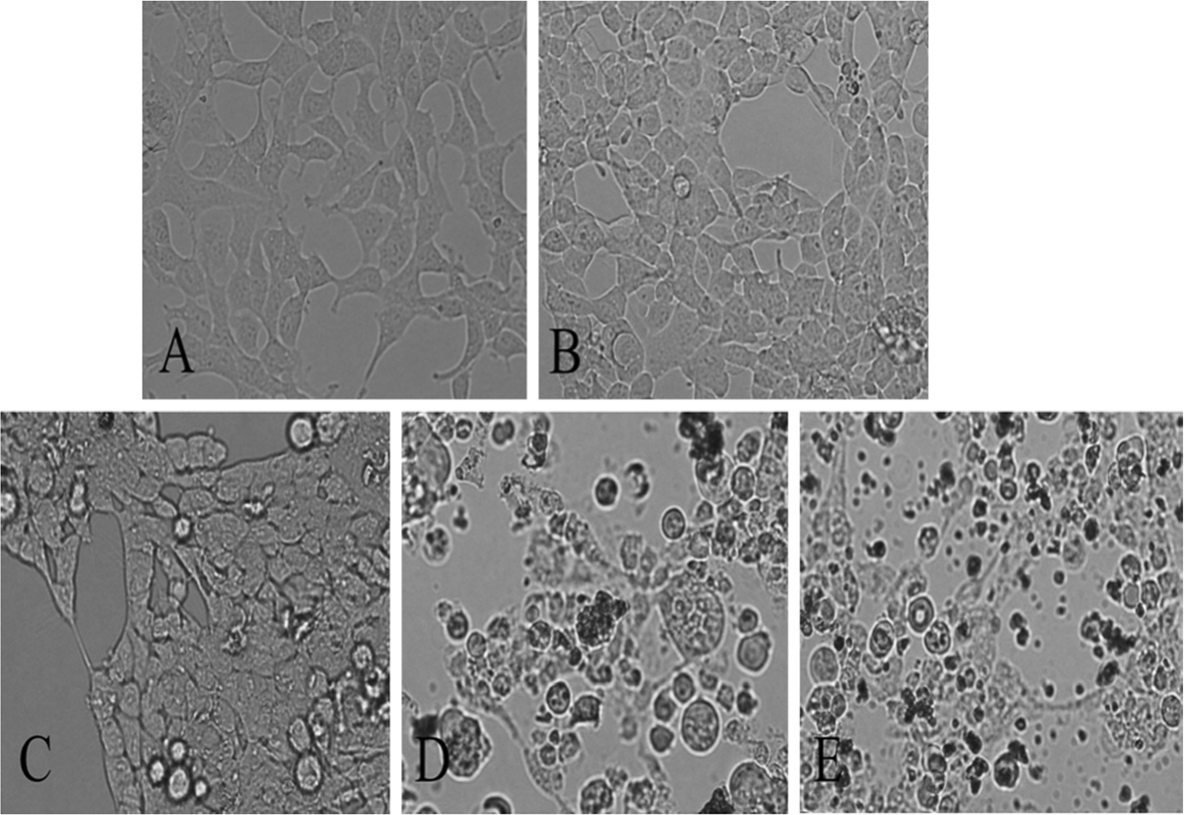
**The morphological changes of HEK293 cells exposed at various concentrations of ZnO NPs for 24 h.** (**A**) normal and non-ZnO NPs-treated, (**B**) 10 μg·mL^−1^, (**C**) 25 μg·mL^−1^, (**D**) 50 μg·mL^−1^, and (**E**) 100 μg·mL^−1^. Magnification ×200.

After L02 and HEK293 cells were incubated with 0, 10, 25, 50, and 100 μg·mL^−1^ of 50 nm ZnO NPs for 24 h, respectively, the resulted cells were observed with 200-fold magnification by optical microscope. L02 cells (Figure 2) and HEK 293 cells (Figure 3) are the comparative morphologies of the unexposed and ZnO particles-exposed cells. Comparing with the bulk cells in the control experiments, the cells cultivated with low dose of ZnO NPs (10 μg·mL^−1^) appear similar to the control cells with brownish particles most likely associated with the cell membranes, indicating that lower dose of ZnO NPs did not harm the L02 and HEK293 cells. With increasing doses of ZnO NPs, the cells started to shrink and became irregular in shape. When the concentration reached 100 μg·mL^−1^, apoptosis, necrosis, and decrease in number of cells were observed. The microscopic studies demonstrated that cells at higher doses became abnormal in size, displaying cellular shrinkage, and acquisition of an irregular shape occurred due to the increase of ZnO NPs concentration.

### Cell activity

**Figure 4:**
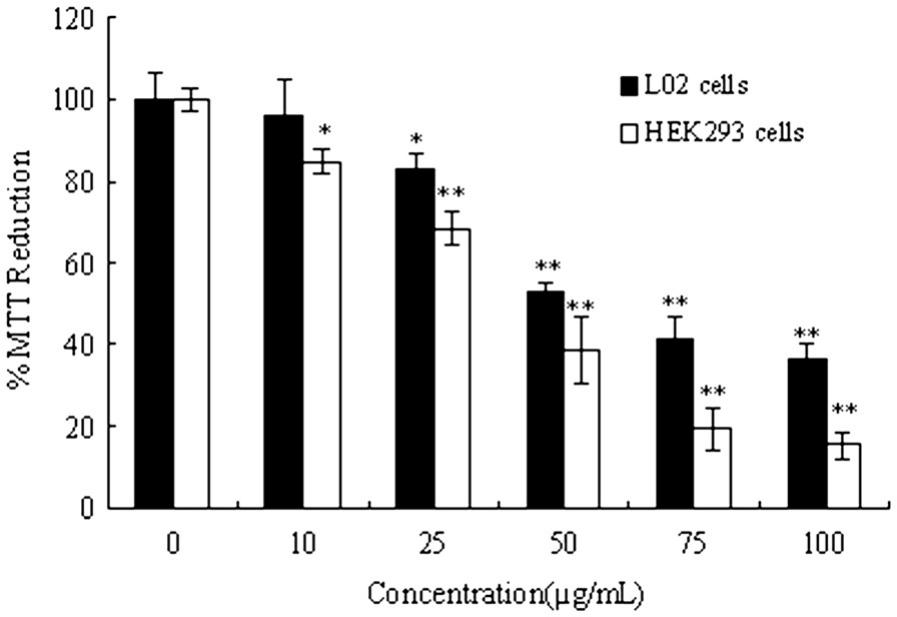
**Cytotoxicity of ZnO NPs on human hepatocyte (L02) cells and human embryonic kidney (HEK293) cells (single asterisk,*p*< 0.05; double asterisk,*p*< 0.01).**

L02 and HEK293 cells were exposed to ZnO NPs (10 to 100 μg·mL^−1^) for 24 h, and cytotoxicity was determined with MTT assay. The MTT results demonstrated a concentration-dependent cytotoxicity after exposure to ZnO NPs (Figure 4). The percentage MTT reduction (relative to control) of L02 cells observed after ZnO exposure at concentrations 50, 75, and 100 μg·mL^−1^ was 52.63%, 41.12%, and 36.70%, respectively; while there were percentage reductions to 68.36%, 38.74%, 19.43%, and 15.21% of HEK293 cells after ZnO NPs exposure at concentrations 25, 50, 75, and 100 μg·mL^−1^. It is observed that there is a statistically significant difference (*p* < 0.05), and the ZnO NPs showed higher toxicity to human embryonic kidney cells.

### Oxidative stress markers

#### Effect of ZnO NPs on malondialdehyde

**Figure 5:**
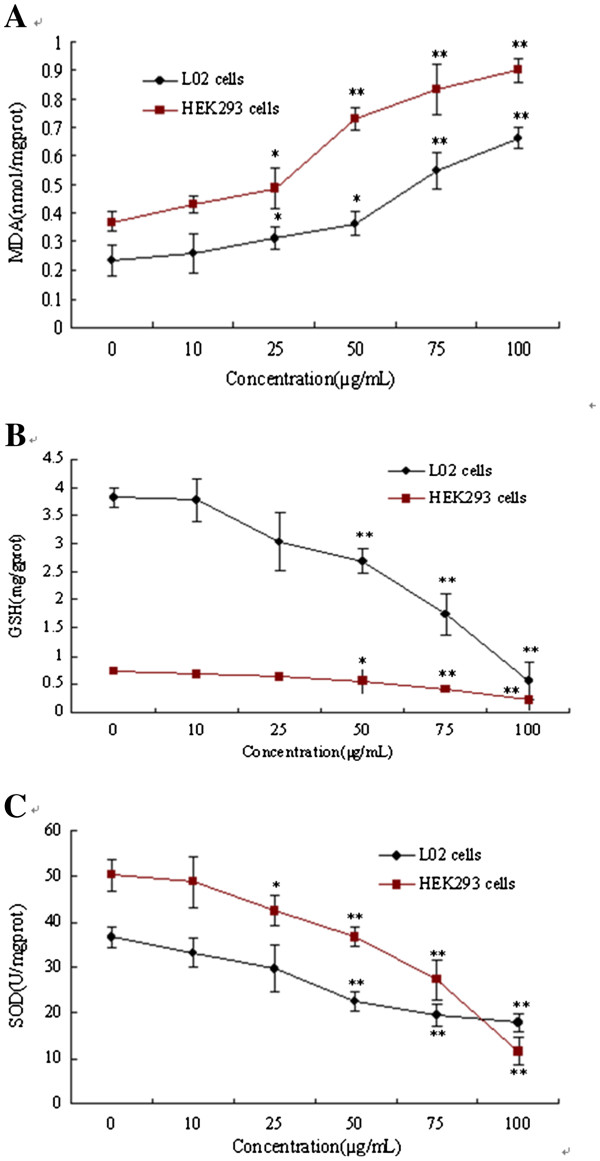
**Effect of ZnO NPs on oxidative stress.** (**A**) Effect of ZnO NPs on MDA. (**B**) Effect of ZnO NPs on GSH level. (**C**) Effect of ZnO NPs on SOD activity (single asterisk, *p* < 0.05 and double asterisk, *p* < 0.01, as compared with the control group).

Lipid peroxidation was examined by measuring MDA concentration. A significant increase (*p* < 0.05) in MDA formation was observed at all concentrations at and above 25 μg·mL^−1^ of ZnO NPs as evident from Figure 5A. It showed that ZnO NPs activated in the cells in a manner of dose-effect relation and had more obvious function to the HEK293 cells (*p* < 0.01).

#### Effect of ZnO NPs on glutathione level

Cells that are exposed to ZnO NPs showed depletion of GSH level in a dose-dependent manner; exposure concentrations exhibiting statistically significant (*p* < 0.05) depletion of 29.66%, 54.43%, and 85.53% for L02 cells and 24.73%, 44.95%, and 70.22% for HEK293 cells at 50, 75, and 100 μg·mL^−1^, respectively after 24 h (Figure 5B).

#### Effect of ZnO NPs on SOD activity

For L02 cells the SOD activity was significantly (*p* < 0.05) reduced at concentrations above 50 μg·mL^−1^ after 24 h of treatment with ZnO NPs when compared to the unexposed cells as evident from Figure 5C. For HEK293 cells the concentrations of ZnO NPs that lead to statistically significant (*p* < 0.05) depletion decreased to 25 μg·mL^−1^.

#### DNA damage

The DNA damage by ZnO NPs was further studied using comet assay. Chromosome abnormalities are the direct consequence of DNA damage such as double-strand breaks and misrepair of strand breaks in DNA, resulting in chromosome rearrangement.

**Figure 6:**
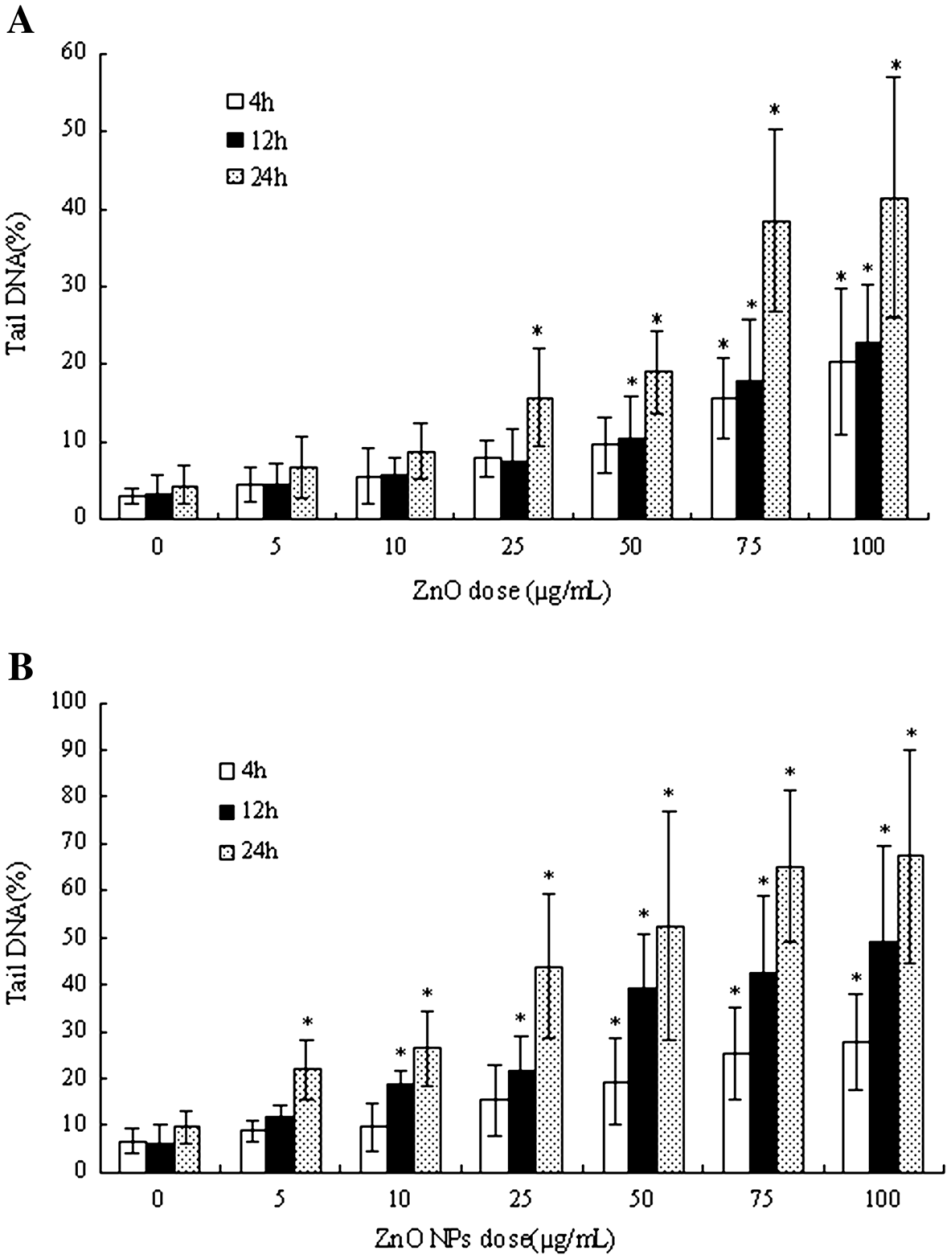
**DNA damage as measured by the comet assay.** DNA damage as measured by the comet assay (percentage of DNA in tail) in human hepatocyte L02 cells (**A**) and human embryonic kidney HEK293 cells (**B**) after exposure to nano ZnO (asterisk, *p* < 0.05).

Comet assay of ZnO NPs-treated cells showed a concentration-dependent increase in tail DNA% as compared to control cells, which gave the extent of DNA damage. In L02 cells ZnO NPs induced a dose-dependent increase in DNA damage after the 4 h treatment, and a significant effect was seen at 75 and 100 μg·mL^−1^. After the 24 h treatment with ZnO NPs, the effect was also clear and a significant increase in DNA damage was observed at tested doses of 25, 50, 75, and 100 μg·mL^−1^, with a significant dependence on dose (Figure 6A).

In HEK293 cells an increase in DNA damage was observed after the treatment with ZnO NPs, and there was a clear dependence on the dose. The 4 h treatment with ZnO NPs induced a significant increase in DNA damage at 50, 75, and 100 μg·mL^−1^, and the effect was dose-dependent. After 24 h treatment with ZnO NPs, the level of DNA damage significantly increased at all tested doses (Figure 6B).

## Discussion

ZnO nanoparticles (ZnO NPs) were previously classified as a new type of high-functional fine inorganic material and have been widely used in consumer and industrial products. However, the cytotoxicity of ZnO NPs has caused wide concerns among scientists and engineers in the last decades [14]. Our results demonstrate that the exposure to ZnO NPs causes morphological changes, cytotoxicity, and oxidative stress to L02 and HEK293 cells. We have also observed the DNA-damaging effects of ZnO NPs on L02 and HEK293 cells for which lipid peroxidation and oxidative stress may be attributed as the probable causes.

The cytotoxicity of ZnO NPs was evident by morphological changes that appeared in two cell lines. The loss of normal morphology started appearing even in 24 h at 25 μg mL^−1^. With a consequent increase in exposure time, the cells retracted into spherical shape and formed clusters in media after detachment from the surface. A high tendency of ZnO NPs adhering to the cell membrane was observed at higher magnification. A previous report suggests that human epidermal cells exposed to ZnO NPs reflect abnormal morphology, cellular shrinkage, detachment from the surface of the flask as well as decreased mitochondrial function, and significantly increased LDH [15,16] at concentrations of 5 to 20 μg mL^−1^ after 24-h exposure [6].

The production of free radicals has been found in a diverse range of nanomaterials, which is one of the primary mechanisms of NPs toxicity [17-20]. It may result in oxidative stress, inflammation, and consequent damage to proteins, membranes, and DNA [6,21-23]. Thus, in our study we investigated the GSH and other antioxidant marker enzymes levels in the cells exposed to ZnO NPs. Depletions in the GSH and SOD level were found on 24-h exposure [24,25]. This indicates a condition of oxidative stress in the cells which may arise due to imbalance in the reactive oxygen species (ROS) formation and antioxidant defense system of the cells [26-28]. As formation of ROS by ZnO NPs is unclear, the mechanism of ROS formation by ZnO NPs needs further investigations.

## Conclusions

A 50-nm ZnO NP was used to culture L02 and HEK293 cells. The results showed that mitochondrial function decreased significantly when exposed to ZnO NPs at 25 μg mL^−1^ in L02 cells and 10 μg mL^−1^ in HEK293 cells. The microscopic studies demonstrated that cells exposed to nanoparticles at higher doses became abnormal in size, displaying cellular shrinkage, and acquired an irregular shape. The GSH and other antioxidant marker enzymes levels in the cells exposed to ZnO NPs were investigated. Depletions in the GSH and SOD level were found on 24 h exposure. With increasing time and dose, DNA damage is more serious, and the migration distance is longer at the same electrophoresis conditions. Our preliminary data suggest that oxidative stress might contribute to ZnO NPs cytotoxicity. To reveal whether apoptosis is involved in ZnO NPs toxicity, further studies are underway.

## Abbreviations

GSH: Glutathione; MDA: Malondialdehyde; MTT: Methyl thiazolyl tetrazolium; SOD: Superoxide dismutase; TEM: Transmission electron microscope.

## Competing interests

The authors declare that they have no competing interests.

## Authors’ contributions

RFG come up with the idea, contribute to the design of the experiment and agreed with the paper's publication; TSK conducted most of experiments that the manuscript mentioned and drafted the manuscript. ZGZ and FL analyzed the data and drew the pictures. HTS and MQL revised manuscript critically and made a few changes. All authors read and approved the final manuscript.

## References

[B1] Gopikrishnan R Zhang K Ravichandran P Baluchamy S Ramesh V Biradar S Ramesh P Pradhan J Hall JC Pradhan AK Ramesh GT Synthesis, characterization and biocompatibility studies of zinc oxide (ZnO) nanorods for biomedical application Nano-Micro Lett 2010 2 31 36

[B2] Zhang YF Zhang B Hu NT Wang YF Wang Z Wang Y Kong ES Poly(glycidyl methacrylates)-grafted zinc oxide nanowire by surface-initiated atom transfer radical poly-merization Nano-Micro Lett 2010 2 285 289

[B3] Suh WH Suslick KS Stucky GD Suh YH Nanotechnology, nanotoxicology and neuroscience Prog Neurobiol 2009 87 133 170 10.1016/j.pneurobio.2008.09.009 18926873 PMC2728462

[B4] Wiench K Wohlleben W Hisgen V Radke K Salinas E Zok S Landsiedel R Acute and chronic effects of nano- and non-nano-scale TiO2 and ZnO particles on mobility and reproduction of the freshwater invertebrate Daphnia magna Chemosphere 2009 76 1356 1365 10.1016/j.chemosphere.2009.06.025 19580988

[B5] Serpone N Dondi D Albini A Inorganic and organic UV filters: their role and efficacy in sunscreens and suncare products Inorg Chim Acta 2007 360 794 802 10.1016/j.ica.2005.12.057

[B6] Hu XK Cook S Wang P Hwang HM In vitro evaluation of cytotoxicity of engineered metal oxide nanoparticles Total Environ 2009 407 3070 3072 10.1016/j.scitotenv.2009.01.033 19215968

[B7] Yuan JH Chen Y Zha HX Song LJ Li CY Li JQ Xia XH Determination, characterization and cytotoxicity on HELF cells of ZnO nanoparticles Colloid Surf B 2010 76 145 150 10.1016/j.colsurfb.2009.10.028 19926459

[B8] Sharma V Shukla RK Saxena N Parmar D Das M Dhawa A DNA damaging potential of zinc oxide nanoparticles in human epidermal cells Toxicol Lett 2009 185 211 218 10.1016/j.toxlet.2009.01.008 19382294

[B9] Berardis BD Civitelli G Condello M Lista P Pozzi R Arancia G Meschini S Exposure to ZnO nanoparticles induces oxidative stress and cytotoxicity in human colon carcinoma cells Toxicol Appl Pharm 2010 246 116 127 10.1016/j.taap.2010.04.012 20434478

[B10] Huang CC Aronstam RS Chen DR Huang YW Oxidative stress, calcium homeostasis, and altered gene expression in human lung epithelial cells exposed to ZnO nanoparticles Toxicol in Vitro 2010 24 44 45 10.1016/j.tiv.2009.09.007 19755143

[B11] Li JY Guo DD Wang XM Wang HP Jiang H Chen BA The photodynamic effect of different size ZnO nanoparticles on cancer cell proliferation in vitro Nanoscale Res Lett 2010 5 1063 1071 10.1007/s11671-010-9603-4 20671778 PMC2893699

[B12] Nair S Sasidharan A Rani VV Menon D Nair S Manzoor K Raina S Role of size scale of ZnO nanoparticles and microparticles on toxicity toward bacteria and osteoblast cancer cells J Mater Sci 2009 20 S235 S241 10.1007/s10856-008-3548-5 18716714

[B13] Lowry O Rosebrough N Farr A Randall R Protein measurement with the folin phenol reagent J Biol Chem 1951 193 265 275 14907713

[B14] Oberdörster G Maynard A Donaldson K Castranova V Fitzpatrick J Ausman K Carter J Karn B Kreyling W Lai D Olin S Monteiro-Riviere N Warheit D Yang H ILSI Research Foundation/Risk Science Institute Nanomaterial Toxicity Screening Working Group: Principles for characterizing the potential human health effects from exposure to nanomaterials: elements of a screening strategy Part Fibre Toxicol 2005 2 28 42 10.1186/1743-8977-2-8 PMC1260029 16209704

[B15] Yang ST Liu JH Wang J Yuan Y Cao AN Wang HF Liu YF Zhao YL Cytotoxicity of zinc oxide nanoparticles: importance of microenvironment J Nanosci Nanotechnol 2010 10 8638 8645 10.1166/jnn.2010.2491 21121377

[B16] Jeng HA Swanson J Toxicity of metal oxide nanoparticles on mammalian cells J Environ Sci Health A 2006 41 2699 2711 10.1080/10934520600966177 17114101

[B17] Nel A Xia T MadleL R Li N Toxic potential of materials at the nanolevel Science 2006 311 622 627 10.1126/science.1114397 16456071

[B18] Lin WS Xu Y Huang CC Ma YF Shannon KB Chen DR Huang YW Toxicity of nano- and micro-sized ZnO particles in human lung epithelial cells J Nanopart Res 2009 11 25 39 10.1007/s11051-008-9419-7

[B19] Deng XY Luan QX Chen WT Wang YL Wu MH Zhang HJ Jiao Z Nanosized zinc oxide particles induce neural stem apoptosis Nanotechnology 2009 20 115101 10.1088/0957-4484/20/11/115101 19420431

[B20] Yang H Liu C Yang DF Zhang HS Xi ZG Comparative study of cytotoxicity, oxidative stress and genotoxicity induced by four typical nanomaterials: the role of particle size, shape and composition J Appl Toxicol 2009 29 69 78 10.1002/jat.1385 18756589

[B21] Bhabra G Sood A Fisher B Cartwright L Saunders M Evans WH Surprenant A Cartwright L Saunders M Evans WH Surprenant A Lopez-Castejon G Mann S Davis SA Hails LA Ingham E Verkade P Lane J Heesom K Newson R Case CP Nanoparticles can cause DNA damage across a cellular barrier Nat Nanotechnol 2009 4 876 883 10.1038/nnano.2009.313 19893513

[B22] Sun C Carpenter C Pratx G Xing L Facile synthesis of amine-functionalized Eu3+-doped La(OH)3 nanophosphors for bioimaging Nanoscale Res Lett 2011 6 24 30 10.1007/s11671-010-9768-x PMC3211300 27502647

[B23] Chen HB Zheng Y Tian G Tian Y Zeng XW Liu G Liu KX Li L Li Z Lin M Huang LQ Oral delivery of DMAB-modified docetaxel-loaded PLGA-TPGS nanoparticles for cancer chemotherapy Nanoscale Res Lett 2011 6 4 13 10.1007/s11671-010-9741-8 PMC3102336 27502629

[B24] Bishop GM Dringen R Robinson SR Zinc stimulates the production of toxic reactive oxygen species (ROS) and inhibits gluatathione reductase in astrocytes Free Radic Biol Med 2007 42 1222 1230 10.1016/j.freeradbiomed.2007.01.022 17382203

[B25] Horie M Nishio K Fujita K Endoh S Miyauchi A Saito Y Iwahashi H Yamamoto K Murayama H Nanashima N Niki E Yoshida Y Protein absorption of ultrafine metal oxide and its influence on cytotoxicity toward cultured cells Chem Res Toxicol 2009 22 543 553 10.1021/tx800289z 19216582

[B26] Liu D Wang LJ Wang ZG Cuschieri A Different cellular response mechanisms contribute to the length-dependent cytotoxicity of multi-walled carbon nanotubes Nanoscale Res Lett 2012 7 361 10.1186/1556-276X-7-361 22748010 PMC3461426

[B27] Ye JP Wang SW Leonard SS Sun Y Butterworth L Antonini J Ding M Rojanasakul Y Vallyathan V Castranova V Shi XL Role of reactive oxygen species and p53 in chromium(VI)-induced apoptosis J Biol Chem 1999 274 34974 34980 10.1074/jbc.274.49.34974 10574974

[B28] Wang YG Aker WG Hwang HM Yedjou CG Yu HT Tchounwou PB A study of the mechanism of in vitro cytotoxicity of metal oxide nanoparticles using catfish primary hepatocytes and human HepG2 cells Sci Total Environ 2011 409 4753 4762 10.1016/j.scitotenv.2011.07.039 21851965 PMC3185176

